# Plant pathogens provide clues to the potential origin of bat white-nose syndrome *Pseudogymnoascus destructans*

**DOI:** 10.1080/21505594.2022.2082139

**Published:** 2022-06-06

**Authors:** Carol Uphoff Meteyer, Julien Y. Dutheil, M. Kevin Keel, Justin G. Boyles, Eva H. Stukenbrock

**Affiliations:** a U.S. Geological Survey, National Wildlife Health Center, Madison, WI, USA; b Molecular Systems Evolution, Max Planck Institute for Evolutionary Biology, Plön, Germany; c School of Veterinary Medicine, Department of Pathology, Microbiology & Immunology, University of California, Davis, Davis, CA, USA; d Cooperative Wildlife Research Laboratory and School of Biological Sciences, Southern Illinois University, Carbondale, IL, USA; e Environmental Genomics Group, Botanical Institute, Christian-Albrechts University of Kiel, Kiel, Germany; f Max Planck Institute for Evolutionary Biology, Plön, Germany

**Keywords:** Dermatophyte, fungal pathogens, hemibiotrophy, hibernation, pathogen emergence, pathogen evolution

## Abstract

White-nose syndrome has killed millions of bats, yet both the origins and infection strategy of the causative fungus, *Pseudogymnoascus destructans*, remain elusive. We provide evidence for a novel hypothesis that *P. destructans* emerged from plant-associated fungi and retained invasion strategies affiliated with fungal pathogens of plants. We demonstrate that *P. destructans* invades bat skin in successive biotrophic and necrotrophic stages (hemibiotrophic infection), a mechanism previously only described in plant fungal pathogens. Further, the convergence of hyphae at hair follicles suggests nutrient tropism. Tropism, biotrophy, and necrotrophy are often associated with structures termed appressoria in plant fungal pathogens; the penetrating hyphae produced by *P. destructans* resemble appressoria. Finally, we conducted a phylogenomic analysis of a taxonomically diverse collection of fungi. Despite gaps in genetic sampling of prehistoric and contemporary fungal species, we estimate an 88% probability the ancestral state of the clade containing *P. destructans* was a plant-associated fungus.

## Introduction

Battling an emerging pathogen requires a thorough understanding of both its origin and the mechanisms by which it attacks the host. Often, these factors are overlooked in the early stages of research on an emerging pathogen as efforts are concentrated on disease surveillance, containment, and possible treatments. This pattern is especially apparent in the study of novel wildlife diseases where resources are limited, and where we lack an understanding of the basic biology of the pathogen and sometimes even the host. Perhaps no wildlife disease better exemplifies this pattern than white-nose syndrome (WNS), a disease with devastating effects on populations of cave-hibernating bats in North America [1]. The causative agent of WNS, *Pseudogymnoascus* (*Geomyces*) *destructans* [2,3], was first isolated over a decade ago, but significant questions still remain about the ultimate origin of this fungus and the mechanism by which it invades the skin of hibernating bats.

**Figure 1.**
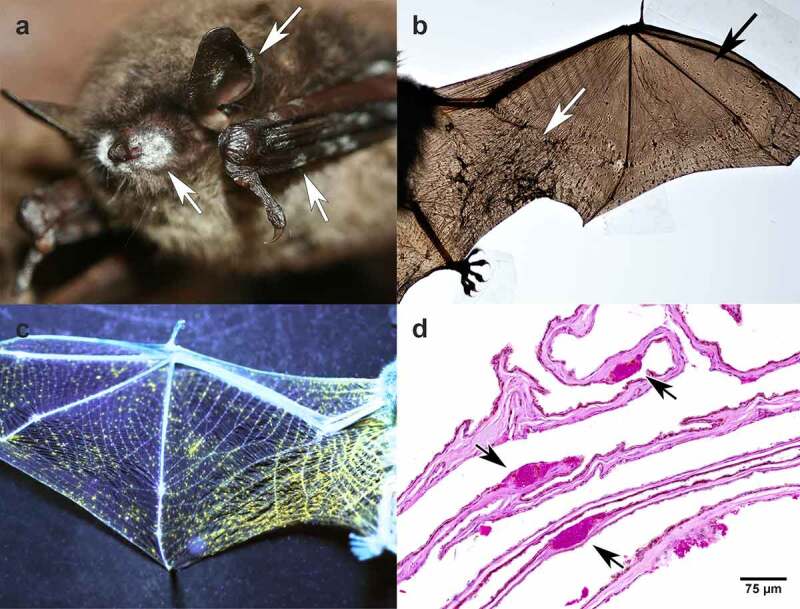
Cave hibernating *Myotis lucifugus* with white-nose syndrome caused by *Pseudogymnoascus destructions*. (a) White patches seen grossly on the muzzle, ear, and wing membrane (white arrows) are aerial hyphae with conidia. Photograph used with permission from Gregory Turner, Pennsylvania game commission, USA. (b) Dark areas of contraction (white arrow) are due to the infection of wing membrane with *P. destructans*. The black arrow points to a region of more normal tissue of the wing membrane. (c) A bat wing illuminated by 385-nm ultraviolet light. The yellow fluorescence corresponds to intraepidermal aggregates of hyphae [4]. (d) Periodic acid Schiff-stained section of wing membrane from a bat with white-nose syndrome. Packets of intraepidermal, magenta-stained hyphae of *P. destructans* (arrows), previously termed ‘cupping erosions’ [5], cause the fluorescence in (c). Absence of inflammatory cell reaction is consistent with immune downregulation that occurs during hibernation.

The colloquial name “white-nose syndrome” (Figure 1(a)) [6] belies the primary site of infection for *P. destructans*: the skin of the wing membrane (Figure 1(b–d)). A visible infection of the muzzle with *P. destructans* is often used as an identifying field characteristic but infection of the muzzle can be absent in the presence of wing infection. An accurate diagnosis of WNS requires the identification of its novel pathognomonic skin lesions, which are best appreciated on the relatively hairless patagium of the wings. These unique and characteristic ‘cupping erosions’ progress to a potentially fatal, invasive, cutaneous ascomycosis as WNS progresses [5].

The invasive nature of *P. destructans* contrasts with the behavior of typical dermatophytes of mammals (e.g. *Trichophyton*, *Microsporum*, *Epidermophyton* spp.), which are superficial parasites, more annoyance than pathogen (e.g. ringworm and athlete’s foot). Normal microflora of human skin, like the yeast *Malassezia*, can infect sebaceous glands and cause superficial infections under certain conditions. Likewise, if the skin surface is otherwise damaged, soil-associated fungi such as *Madurella* spp. and *Pseudogymnoascus* (*Geomyces*) *pannorum* can secondarily infect these penetrating wounds and “digest” animal tissue. Other fungal infections occur in hibernating bats that can cause patches of white fungal growth on skin that grossly resemble WNS, but histologically resemble typical dermatophytes with infection limited to the keratin layer of the skin without invasion [7]. The important distinction is that, on their own, these dermatophytes cannot invade an intact skin surface. Indeed, most fungi that can cause pathology in animals exist primarily as saprobes and are opportunistic pathogens that do not require an animal host [8]. *P. destructans* is unique among known mammal-associated cutaneous fungi in its ability to actively penetrate the normal, intact skin of its host.

Several other characteristics distinguish *P. destructans* from typical dermatophytes. *P. destructans* is an obligate psychrophile that inhabits the consistently cold hibernacula where bats overwinter [9], while other dermatophytes tolerate the consistently high body temperatures of homeothermic mammals. Further, dermatophytes are nourished by non-living keratin on skin surface, which they access through secretion of keratinase [10]. *P. destructans* has not been shown to hydrolyze keratin [11,12]. Instead, it produces lipases, proteinases, endopeptidases, collagenase, esterase, hemolysins, urease, and chitinase [11,13], pointing to a different nutrition strategy because these enzymes hydrolyze fat, protein, collagen, etc. Finally, it appears that *P. destructans* undergoes morphological shifts not seen in typical mammal dermatophytes during invasion. When found on the surface, hyphae of *P. destructans* are delicate with parallel walls and are non-fluorescent when exposed to longwave ultraviolet (UV) light. When found in the diagnostically characteristic cupping erosions, the hyphae change morphology and are wide and bulbous, with irregular non-parallel walls [5]. Here, the distinct bundles of hyphae fluoresce under UV light (Figure 1(c,d)) [4]. The change in fluorescence is considered evidence of a physiological shift [14], which is seen in fungal plant pathogens as invasion of their plant host progresses through various stages [15]. Although *P. destructans* has been considered an unusual dermatophyte that was recently introduced to North America with devastating effect on hibernating bats, previous research has not made an explicit attempt to address the unusual pattern of skin invasion or the origin of *P. destructans*. Evidence would indicate it is unlikely *P. destructans* evolved from a typical mammalian dermatophyte.

Proteins associated with *P. destructans* sampled under different environmental conditions [16] provide evidence that *P. destructans* may have originated from an environmental saprophyte. However, although it can survive in sediments when bats are not present, *P. destructans* is outcompeted by other fungi commonly found in hibernacula [17,18]. This is consistent with findings that *P. destructans* is less efficient than sympatric fungi at metabolizing plant derived cellulose substrates and remains of other microbes and invertebrates, including chitin, that are a primary nutrient sources for saprophytes [13]. In addition, a comparative genome study reported a recent duplication of the high affinity nitrate transporter *NRT2* in *P. destructans* that may reflect a more recent enhancement of *P. destructans’* ability to use environmental nitrogen [19]. These findings suggest that *P. destructans* is unlikely to be a primary saprophyte acting as an opportunistic pathogen, but rather a primary pathogen and an opportunistic saprophyte. However, none of this evidence rules out contributions from an ancestral environmental fungus, particularly an obligate cryophile, in the evolution of *P. destructans*.

Previous efforts to characterize *P. destructans* have left the question of its origins unanswered. Over the course of examining more than a hundred WNS-positive bats, we first recognized an unusual mechanism of skin invasion by *P. destructans*, previously undescribed in mammalian pathogens, and further recognized that invasion mechanisms might provide clues about the ultimate origin of *P. destructans* (C. Meteyer, U.S. Geological Survey, personal observation, [2008–2012]). Here, we detail these histopathological findings and add transmission electron microscopy and phylogenomic analyses to more explicitly address both the invasion mechanism and ultimate origins of *P. destructans*. These multiple lines of independent evidence point to plant-associated fungal pathogens as ancestors of *P. destructans*.

## Results and Discussion

**Figure 2.**
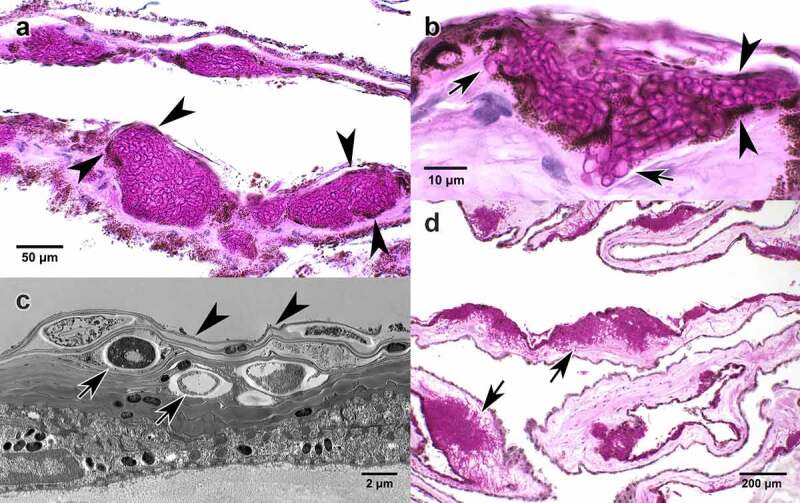
Wing membrane. Histologic periodic acid Schiff stain (a, b, d) and transmission electron micrographic (TEM, c) sections of wing membrane from *Myotis lucifugus* infected with *Pseudogymnoascus destructans* causing white-nose syndrome. Fungal hyphae are stained magenta. Lack of inflammatory cell reaction is consistent with WNS in hibernating bats [5]. (a) Pigmented cells of the epidermal layer (arrowheads) illustrate the intraepidermal boundary above and below dense packets of irregular *P. destructans* hyphae without epidermal damage [21]; this is consistent with the biotrophic stage of invasion. (b) Intraepidermal proliferation of fungal hyphae demarcated above and below by pigmented epidermis (arrowheads). Arrows show hyphae penetrating the basement membrane of the epidermis initiating infection of the deeper dermis. This hyphal invasion resembles the penetration mechanism used by some types of appressoria. (c) TEM shows fungal hyphae (arrows) within the outer layer (stratum corneum) of the epidermis. Arrowheads indicate the layer of bat epidermal cells covering hyphae without morphologic change or evidence of damage to the host cells; this is consistent with biotrophic infection. (d) Progression of *P. destructans* infection into deeper dermis (arrows) with replacement of connective tissue of the wing membrane with fungal hyphae.

To investigate mechanisms of bat skin invasion by *P. destructans*, we analyzed microscopic details of the hyphae as they invaded wing membranes and muzzles in naturally infected bats. Our microscopic and electron microscopic studies of wing tissue elucidate two distinct stages of infection as *P. destructans* invades the skin of bats. The first stage of infection forms the epidermal bundles of irregular hyphae, previously termed “cupping erosions” (Figure 1(d)), that are so unique among fungal skin infections they continue to be the gold standard for diagnosing WNS, even with the development of molecular diagnostic tests [5,20]. Importantly, our studies reveal the cupping erosions are not actual erosions in the skin surface, but proliferation of hyphae within the intraepidermal space, confined above and below by epidermal cells without associated damage to the epidermis (Figure 2(a–c)). This initial stage of *P. destructans* infection is morphologically similar to the biotrophic strategy of infection used by fungal pathogens of plants [22]. Biotrophic infection, the propagation of an invading organism within living host tissues without causing tissue damage, has been extensively studied in plant fungal pathogens; it has not been detailed as a strategy of fungal infections in animals.

As infection progresses, *P. destructans* invades beyond the epidermis into deeper layers of skin. This second stage of infection is initiated by hyphae that penetrate the basement membrane that separates the epidermis and dermis (Figure 2(b)). After penetration, hyphae proliferate in the dermis, hydrolyzing and replacing host tissue (Figure 2(b,d)). This stage of infection is similar to the necrotrophic strategy of plant fungal pathogens. Hemibiotrophic species, those that switch from biotrophic infection to necrotrophic infection, are well studied among plant pathogens [23] but are unknown among fungal pathogens of vertebrates, including mammals. Invading hyphae seen in histologic sections of bats infected with *P. destructans* (Figure 2(b)) resemble appressoria, which have also been well described in plant fungal pathogens. Appressoria represent a category of structures used for host invasion. They vary in morphology and mechanism, but have previously not been documented in fungal pathogens of mammals [24].

Another well-known wildlife disease that has contributed to population declines, chytridiomycosis, serves as an informative contrast to demonstrate the uniqueness of *P. destructans*’ invasion strategy among vertebrate fungal pathogens. As *P. destructans* invades deeper layers of skin as it spreads along the wing membrane in hibernating bats, the disruption of the epidermis disturbs thermoregulation, water and electrolyte balance, and respiration, potentially leading to death [25,26]. The causative fungal pathogen of chytridiomycosis, *Batrachochytrium dendrobatidis*, also compromises the critical respiratory function of the skin in many species of frogs, but remains in the epidermis and does not invade the deeper dermis. This primitive, single-celled, non-filamentous fungus uses keratin as its nutritional source, encysts on the surface of the skin and develops a germ tube that penetrates a host cell to generate immature sporangia [27]. As a surface infection, *B. dendrobatidis* survives on the superficial non-living keratin layer of the host which thickens in response to infection. As the keratin layer thickens (hyperkeratosis), the osmoregulation and respiratory functions of the amphibian skin is disrupted and can eventually lead to death [27]. Thus, even though *P. destructans* and *B. dendrobatidis* lead to death through disruption of the physiology of the skin in their respective hosts, the infection strategies and the resulting pathology are very different.

**Figure 3.**
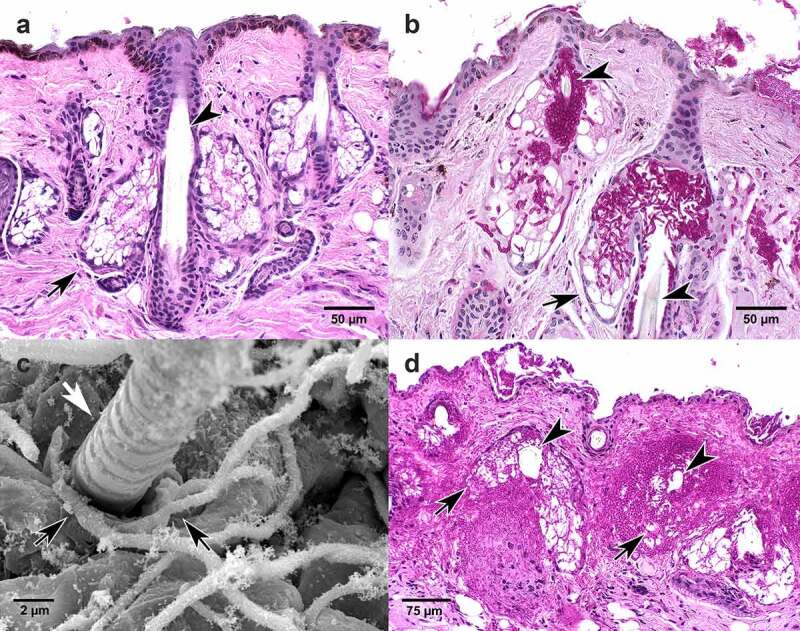
Muzzle. Histologic sections of muzzle from *Myotis lucifugus* stained with hematoxylin and eosin (a), periodic acid Schiff stain (b, d), and transmission electron micrographic (TEM, c). (a) Normal muzzle tissue stained with hematoxylin and eosin. Arrowhead points to hair shaft. Black arrow points to sebaceous gland that secretes lipid material into the hair follicle. (b) Fungal hyphae of *Pseudogymnoascus destructans* (magenta) within and expanding from the follicle (arrowheads), invading and replacing lipid-laden sebaceous gland (arrow) and surrounding connective tissue. (c) TEM shows fungal hyphae (black arrows) entering the hair follicle identified by emerging hair shaft (white arrow). (d) Extensive invasion of *P. destructans* including hair follicle surrounding hair shaft (arrowheads) with replacement of associated sebaceous glands (arrows) and regional connective tissue.

Our microscopic and electron microscopic studies also provide evidence that the hyphae of *P. destructans* seem to detect and respond to nutritional sources similar to nutrient tropism in fungal pathogens of plants and insects. In WNS, *P. destructans* produces hyphae and conidia that cover the muzzle and create the titular “white nose”. The haired skin of the muzzle is endowed with abundant sebaceous glands [28], which produce simple and complex lipids secreted through the sebaceous pore into the hair follicle (Figure 3(a)). In addition to intraepidermal invasion of the muzzle, similar to what occurs in tissues of the wing membrane, the hyphae of *P. destructans* also invade and replace sebaceous glands as the enzymes it produces (e.g. lipase, esterase, and proteinase) hydrolyze lipids, fatty acids, cholesterol esters, and triglycerides produced by these glands (Figure 3(b)). Many fungal pathogens of plants and insects have evolved nutrient sensing and signaling pathways [29]. When these pathways are activated they induce growth of hyphae toward the nutrient source (tropism) and initiate the differentiation of specialized structures to access nutrients, including pseudohyphae, hyphal extensions, and appressoria [29]. The term appressoria encompasses a wide range of morphologies and invasion mechanisms [30–34]. Although appressoria or appressoria-like structures are widely described as crucial infection structures of some fungal pathogens of plants and insects [35,36], they have not been identified in vertebrate pathogens [24].

Our transmission electron microscopic images of bat muzzles infected with *P. destructans* point to a similar tropism-induced growth of *P. destructans* hyphae toward a lipid nutrient source (sebaceous gland) as they are consistently seen at the openings of hair follicles (Figure 3(c)) and within sebaceous glands in bats with “white noses”. In this nutrient rich environment, hyphae proliferate, hydrolyzing and obliterating regional tissue (Figure 3(b,d)). In addition to sebaceous glands, the epidermis covering the muzzle and wing membrane is also rich in lipids that bathe surface corneocytes in a complex extracellular lipid matrix [37]. We propose that detection of lipids and other nutrients available in the intraepidermal spaces of bat skin and in sebaceous glands activates a similar nutrient signaling pathway in *P. destructans* that might lead to the formation of appressoria. Our transmission electron microscopic images were limited and did not allow us to conclusively characterize appressoria, but invading hyphae seen in histologic sections (Figure 2(b)) are consistent with appressoria. Further *in vitro* and *in vivo* infection studies would be valuable to determine whether or not appressoria are induced in *P. destructans* to invade host tissues. A transcriptome study of *P. destructans* from infected bat wing membrane identified genes encoding enzymes involved in the remodeling of fungal cell walls [38]. Cell wall remodeling is not specific to appressoria formation but it is necessary for their development [39]. If confirmed, the development of appressoria or appressoria-like structures would provide additional evidence for the use of plant pathogen invasion strategies in a mammalian host.

**Figure 4.**
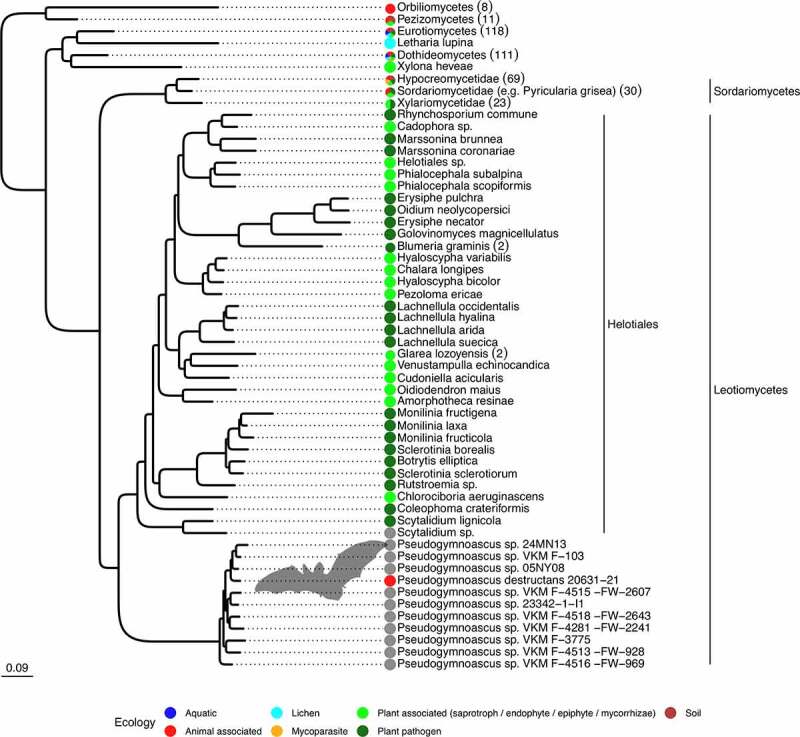
*Pseudogymnoascus* species are phylogenetically related to plant-associated fungi. Maximum likelihood tree based on 1,146 families of orthologues. All displayed nodes are supported by a bootstrap value >95%. The number of sequences in each collapsed clade are indicated within parentheses. The tree is represented in its more complete form inFigure S-1. Within the Leotiomyceta clade, groups outside *Pseudogymnoascus* have been collapsed at the species level, with the corresponding number of strains indicated in parentheses. Color dots indicate known ecologies. The Leotiomyceta clade does not contain any representatives of the ecology groups for aquatic, lichen, mycoparasite of plant pathogen, or mammalian pathogens outside of the *Pseudogymnoascus* genus. Additional species have been discarded because they were closely related to species with more sequence data, a list of which can be found in Supplementary Table S1.

To investigate the origin of *P. destructans* more thoroughly in light of its unique characteristics, we conducted a high resolution phylogenomic analyses of a broad inventory of ascomycete species. As previously shown in a more taxonomically focused phylogenomic reconstruction [40], *Pseudogymnoascus* forms a well-supported sister group to the order Helotiales within the class Leotiomycetes, but with a long branch illustrating the relatively poor sampling of this lineage. To expand on this analysis, we broadened taxonomic coverage, making use of publicly available sequence data, and further collected information about the “lifestyle” associated with taxa for which information was available. The resulting phylogeny demonstrates the uniqueness of *P. destructans* in terms of host range among closely related species. The class Leotiomycetes is highly enriched with species known to be associated with plants in different ways: as pathogens, endophytes, or saprotrophs (e.g. *Coleophoma* spp., *Cudoniella* spp. and *Lachnellula* spp.) [41,42] (Figure 4). For example, among the genera in the clade with *P. destructans* is *Botrytis* spp., which contain important plant pathogens [43]. Using phylogenetic models of character evolution, we quantified the probability that the ancestral state of the clade containing *P. destructans* was associated with plants (as a pathogen or not) to be at least 88% (Table S-2).

Collectively, our morphologic and phylogenomic analyses provide evidence for our hypothesis that *P. destructans* evolved from an ancestor that was adapted to coexist not with mammals but with plants, with invasion strategies that resemble hemibiotrophic plant pathogens. In a different context, the similarity of *P. destructans* to plant pathogens was demonstrated by Pannkuk and colleagues [44] who investigated an extracellular subtilisin-like serine protease produced by *P. destructans*. Although subtilisin serine proteases are produced by typical dermatophytes and promote skin adhesion during infection [10], comparative amino-acid sequence analyses showed that the serine protease produced by *P. destructans* has a higher similarity to serine proteases of diverse fungal plant pathogens including *Sclerotinia* and *Botrytis* than dermatophyte pathogens like *Arthroderma*, *Trichophyton*, and *Microsporum* [44].

Although *P. pannorum*, a widespread soil saprophyte, is considered a close contemporary to *P. destructans*, it was not included in our final phylogenomic analysis because of insufficient data (see methods for details). The comparison between these two fungi is interesting because of the close (presumed) relationship but pronounced physiological differences. *P. pannorum* is broadly thermotolerant allowing it to infect homeothermic mammals, including humans [45]. It cannot, however, independently invade animal tissue, instead requiring preexisting damage to the skin for infection to occur. Conversely, *P. destructans* uses a novel approach that allows it to invade the intact skin of mammals while being restricted in its host range as an obligate psychrophile. Comparisons of these congeners may lead to a better understanding of the evolutionary history of the clade as a whole and may offer clues about the unique confluence of conditions that could facilitate a shift from plant-associated fungus to a cryophilic mammalian pathogen.

We can currently only speculate about what might have transpired millions of years ago that allowed *P. destructans* to adapt as a pathogen of hibernating bats using strategies that resemble those of plant pathogens, but there are hints in the literature as to where such an investigation might begin. A previous study comparing the evolutionary genomics of Eurasian bats and *P. destructans* suggests their ancestors could have temporally overlapped with the potential to co-evolve during the Cenozoic [40]. During this era, Eurasia (and indeed most of the planet) would have been in the middle of the Cenozoic cooling, characterized by decreasing annual temperatures and increasing seasonality [46,47]. Such a climate would necessitate the evolution of strategies such as hibernation to allow mammals to survive cold periods when food was not available. A gradual shift to hibernation across geological time [48] might have provided plant-associated ancestors of *P. destructans* an opportunity to intermittently and progressively adapt to a new host that was becoming thermally compatible for longer and longer periods as the body temperature of hibernating bats dropped within the ideal temperature range for *P. destructans* [9]. Further, finding refuge in consistently cool caves to hibernate and avoid freezing could have brought bats in proximity to a cryophilic fungus like *P. destructans*. There are other possibilities, including the jump to hibernating bats through an intermediate host with labile body temperature, like seen in some extant tropical and subtropical mammals to this day [49]. Still, the simplest explanation, and one warranting further exploration is the co-evolution of bats and *P. destructans* as bats adapted from mild to progressively more severe winters. Initially intermittent infections of short duration might have provided evolutionary pressure over a geological timescale for ancestors of *P. destructans* to progressively adapt virulence strategies to take advantage of hibernating bats as a new host niche. Initial infections of short duration during bouts of torpor might also have allowed ancestral bats to develop tolerance to *P. destructans*. Comparing immune effector proteins from populations exposed to *P. destructans* for different lengths of time [50,51] provides additional contemporary evidence for adaptation/tolerance and co-evolution.

This evolutionary scenario also provides a hypothetical explanation for the mild infections seen in Eurasian bats infected with *P. destructans* when compared to the severity of infections and death experienced by North American bats [52]. Genetic diversity is higher among Eurasian isolates of *P. destructans* than North American isolates showing a longer evolutionary history in Eurasia and recent introduction with clonal expansion in North America [3,53,54]. North American *P. destructans* isolates are more closely related to European isolates, whereas the genomes of isolates from these geographic regions are distinct from those isolated in China, supporting the introduction of *P. destructans* from Europe to North America [53].

Geographical isolation of North American bats, and their recent ancestors, from *P. destructans* was breached with a recent transcontinental introduction of this unique pathogen [53]. With patterns of host skin infection that resemble those of a plant pathogen, and recent introduction into North America, it would be expected that North American bats would have none of the tolerance found in Eurasian bats [55–57]. Conversely, *P. destructans* would be perfectly adapted to infect and cause severe disease in North American bats. The result has been a predictable and rapid spread of *P. destructans* in hibernating bats across the continent, and unprecedented mortality associated with WNS [1]. A similar scenario might have played out at least once before as well because mass accumulations of bat skeletal remains in Europe suggest a significant mortality event during the Pleistocene and Pliocene (5.4 million years ago − 11,700 years ago) that might have been associated with the introduction of a novel pathogen, proposed to be the initial introduction of *P. destructans* into Europe [52].

Our study used multiple lines of evidence to provide a novel hypothesis about the origin of *P. destructans*. Specifically, we propose that *P. destructans* is adapted from plant-associated fungi, with invasion strategies compatible with ancestral contributions from plant pathogens. Importantly, the plant-pathogen hypothesis is testable. For example, transcriptome analysis can characterize significant physiological stages of invasion such as the gene transcriptome that functions as the “switch” from a biotrophic to necrotrophic infection [23]. Similarly, *in vitro* systems exist for real-time imaging of invasion by fungal plant pathogens, including fluorescent contrast studies to track appressoria [39]. Similar imaging and transcription studies could be applied to *P. destructans*.

We have proposed a novel, testable hypothesis that could act to bridge research between scientists studying the pathogenesis of plant fungi and scientists studying the pathogenesis of animal fungi. Research on WNS started from nothing about 15 years ago, but plant fungal pathogens have been the subject of intense research for decades. A trove of knowledge has been accumulated from plant-based research that might be applicable to WNS in bats. Taking advantage of this accumulated knowledge and recruiting scientists from the field of fungal pathogenesis in plants can accelerate the understanding of *P. destructans* and its novel host-pathogen relationship while potentially providing new paths to prevent WNS. Applying standard tools used in the study of plant fungal pathogens to *P. destructans* also has the potential to add a new model system to better understand mechanisms virulent fungal pathogens use to invade plant hosts. And finally, bridging research between scientists working with plant and animal pathogens will almost certainly lead to new approaches and insights into how new pathogens emerge from physical and biological environments and adapt to new hosts, including those in distant kingdoms.

## Materials and methods

### Microscopy

Histopathology that contributed to this paper included wing samples taken after full necropsy and examination of wings of 168 bats (47% positive for intraepidermal fungal proliferation without necrosis or inflammation – the hallmark of WNS) of 11 species (60% *Myotis lucifugus*, 16% *Perimyotis subflavus*) submitted to the U.S. Geological Survey (USGS) National Wildlife Health Center in Madison, Wisconsin, USA from 21 states between March 2009 and April 2012 [4]. More details regarding the diagnostic findings for these bats can be found in [13], including Table S1. We conducted histological and microscopic observations on bats submitted to the USGS National Wildlife Health Center for diagnosis and documentation of WNS. We followed standard protocols for histology [58]. Briefly, we fixed sections of skin in 10% neutral buffered formalin, embedded them in paraffin, cut them to a thickness of 5 microns, placed them on glass slides, and stained with the periodic acid-Schiff (PAS). To conduct transmission electron microscopy, we fixed tissues in 10% neutral buffered formalin and then post-fixed samples of the patagium in glutaraldehyde. We embedded tissues in epon embedding resin and poststained thin sections with saturated aqueous uranyl acetate and Reynold’s lead citrate. Finally, we collected images with a 200kV JEOL 2100 LaB6, transmission electron microscope (Peabody, Massachusetts).

### Phylogenomic analysis

We retrieved the protein gene catalog of *P. destructans*, version 20170919, from Mycocosm, the Joint Genome Institute (JGI) fungal genome database (https://mycocosm.jgi.doe.gov/Pseudest1/Pseudest1.home.html) [59]. The complete proteome was then used as a query for a homology search in the National Center for Biotechnology Information (NCBI) non-redundant (nr) protein database. For computational efficiency, we downloaded the complete nr dataset from the NCBI server (http://ftp.ncbi.nlm.nih.gov/blast/db/FASTA/nr.gz) and used the DIAMOND program as a rapid alternate to BLAST [60]. We structured the nr dataset into a DIAMOND database including taxonomic information, and then conducted the homology search using the “blastp” algorithm of DIAMOND, with the options -k0 (to report all significant alignments for each query sequence), -e 1e-6 (significance threshold of 1e-6 for reporting an alignment), -b4 (block size of 4, as automatically suggested by the program based on the computer configuration), and – taxonlist 4751 (to limit the search to fungal sequences). The search results were generated in tabular format, and we further split them per query sequence to facilitate downstream analyses. In cases where multiple high scoring pairs (HSP) were found for a reference sequence, we only retained the most significant one. All scripts and data are available at https://gitlab.gwdg.de/molsysevol/pseudogymnoascus-destructans-phylogeny/.

We then reconstructed the presence-absence pattern of each one of the 9,310 *P. destructans* genes using the taxonomic information produced in the DIAMOND database. Our database search revealed homologous sequences in 36,578 fungal species, so we generated a 9,310 x 36,578 table containing the number of homologous sequences found in each species for each gene. In this context, a table entry of zero indicates the gene was not found in a particular species, either because it does not have a homologous sequence (i.e. the corresponding homolog was not sequenced) or because the sequence was too divergent to lead to a similarity score higher than the threshold used for significance assessment.

As we are interested in speciation and not duplication events, we discarded paralogous sequences, in particular to minimize the problem of hidden paralogy. For each species, we set the occurrence number of the genes that were present in multiple copies to zero in the matrix. In order to minimize the final amount of missing data, we first only retain species that had at least 1,000 genes with an identified homolog in *P. destructans*, keeping a total of 1,257 species. A lack of quality sequence data for the congener, *Pseudogymnoascus pannorum*, precluded its inclusion beyond this step in this analysis (the cause of “snake fungal disease”, *Ophidiomyces ophiodiicola*, also lacked adequate sequence data to be included). Second, we kept only genes present in at least 80% of all species, reducing the number of genes to 1,146.

We next retrieved the amino-acid sequences of the selected species for each of the 1,146 selected genes. We aligned each family dataset using two distinct methods: Muscle [61] and Clustal Omega [62]. We compared the two resulting alignments using the bppAlnScore program from the Bio++ Program Suite [63], and only selected positions recovered identically by the two methods for downstream analyses. We ran all analyses in parallel using the GNU parallel software [64]. All 1,146 filtered alignments were concatenated, leading to a total alignment of 461,596 amino-acid positions.

We conducted a phylogenetic analysis in several steps because the large data matrix prohibited the inference of a maximum likelihood tree in a reasonable time. First, we used FastTree [65] and the complete data set to build an initial maximum likelihood tree using the Le and Gascuel substitution model [66]. In order to run a more thorough search using the RaxML-NG software [67], we reduced the dataset by removing sequences that are highly similar. Using the PhySamp program [68], we sampled sequences based on their similarity: when two sequences were more than 97% identical, the one with the lowest amount of missing data (unresolved character or gap) was kept and the other sequence discarded. The resulting similarity clusters and their representative members are listed in Supplementary Table S1, and the final sampled tree included 817 species. We used RaxML-NG software [67] to estimate the phylogeny from the concatenated alignment by maximum likelihood (ML), following the protocol described in [69]. We used an LG model of amino-acid substitutions [66] with a four-classes gamma distribution of rates. We used the FastTree tree as starting tree and compared two models: one where all 1,146 genes followed the same model (1,632 parameters), and a partition model where each gene was allowed to have its own rate distribution and a distinctive branch scaling factor (3,922 parameters). This latter model led to a lower Akaike Information criterion (AIC) (397,373,191 vs. 398,450,754) and was selected for downstream analyses. The resulting tree is plotted in Supplementary Figure S1. We then focused on the Pezizomycotina clade. We pruned the subtree from the RaxML tree and selected the corresponding sequences. We then conducted a more thorough ML inference on this sub-dataset, using 20 distinct initial trees (10 random and 10 obtained by maximum parsimony) and retained the resulting estimated tree with the highest likelihood. To obtain support values for all branches in the tree, we further conducted a bootstrap analysis with 100 replicates, which was sufficient to get reliable bootstrap values based on the extended majority rule (MRE)-based bootstrapping test implemented in RaxML [70]. We plotted the resulting phylogeny using the ggtree package for R [71]. The data that support this study are openly available in pseudogymonoscus-destructans-phylogeny at https://gitlab.gwdg.de/molsysevol/

### Life history trait annotations

We classified each species as one of the following: animal associated (including nematophagous, pathogen, coprophilous), plant associated (including saprotroph, endophyte, epiphyte, mycorrhizae), plant pathogen, mycoparasite, lichen, aquatic, soil. We first attempted to assign classifications based on descriptions from the JGI fungal genomics database. For species not well described in the database, we conducted a general literature search, and recorded species as missing data when no reliable information could be found. Many fungal species are associated with multiple environments or organisms, so we conservatively recorded a pathogen as animal associated if there is a record of any animal infection. All assigned life history traits are available in Figure S1.

### Ancestral trait reconstruction

We inferred the characteristics of the *P. destructans* ancestor using models of discrete character evolution. We used the Pezizomycotina maximum likelihood tree and discarded species for which no life history trait data could be found in the literature. The resulting dataset contained 355 species and seven character states: “animal associated” (87 species), “aquatic” (7 species), “lichen” (3 species), “mycoparasite” (4 species), “plant associated” (114 species), “plant pathogen” (121 species) and “soil” (19 species). Because some character states were observed in low frequency, we also analyzed a reduced dataset restricted to the most frequent states: “animal associated”, “plant associated”, “plant pathogen” and “soil,” covering 341 species. We fit three models of character-state transition to each dataset: all changes equally likely (equal rate, ER), all rates different but symmetric changes equally likely (symmetric model, SYM), and all rates different (ARD). We used the “ace” function of the ‘ape’ package for R [72] to fit models and calculate Akaike’s information criterion (AIC) for each model. To estimate the character state of the *P. destructans* ancestor, we used a stochastic mapping procedure [73,74] as implemented in the ‘phytools’ package for R [75]. We sampled 1,000 maps of character changes from the posterior distribution, accounting for the uncertainty in the substitution matrix (argument Q = “msmc” in the ‘make.simmap’ function). We used the ‘describe.simmap’ function for ancestral state estimation of each model and the ‘aic.w’ function and AIC to average models (Supplementary Table S2). Scripts used for the analyses are openly available in https://gitlab.gwdg.de/molsysevol/pseudogymnoascus-destructans-phylogeny.

## Supplementary Material

Supplemental Material

## Data Availability

The data that support the findings of this study are openly available in Zenodo at DOI reference number 10.5281/zenodo. 5939906 at https://zenodo.org/record/5939906#.YjpiN-rMKUk.
