# Supplementary material for: Plant pathogens provide clues to the potential origin of bat white-nose syndrome Pseudogymnoascus destructans
Source: Virulence. 2022 Jun 6;13(1):1020–31. doi: 10.1080/21505594.2022.2082139 (PMC9176227; doi:10.1080/21505594.2022.2082139)
Supplement: Supplemental Material [file KVIR_A_2082139_SM3848.zip › supplementary/Revision Supplementary Table S1.pdf]

Table S1. Species sampling based on sequence similarity. Species less than 3% divergent at the protein sequence level were sampled to keep the longest representative.

	SuperAlignment3.Sampled03.clust
**Representative species**	**Species removed[1]**
Komagataella_phaffii_CBS_7435	Komagataella_phaffii_GS115
Stachybotrys_chartarum_IBT_7711	Stachybotrys_chlorohalonata_IBT_40285
Penicillium_rubens	Penicillium_nalgiovense
Fusarium_pseudograminearum_CS3096	Fusarium_venenatum,Fusarium_poae,Fusarium_langsethiae
Pyricularia_oryzae_P131	Pyricularia_oryzae,Pyricularia_oryzae_70-15
Trichoderma_reesei_RUT_C-30	Trichoderma_parareesei
Blastomyces_dermatitidis_ATCC_26199	Blastomyces_dermatitidis_ATCC_18188
Penicillium_digitatum_PHI26	Penicillium_digitatum_Pd1
Fusarium_fujikuroi_IMI_58289	Fusarium_oxysporum_f._sp._cubense_race_1,Fusarium_oxysporum_Fo5176,Fusarium_oxysporum_f._sp._narcissi, Fusarium_gaditjirri
Metarhizium_brunneum	Metarhizium_majus_ARSEF_297,Metarhizium_guizhouense_ARSEF_977
Monosporascus_sp._MC13-8B	Monosporascus_cannonballus
Talaromyces_marneffei_ATCC_18224	Talaromyces_marneffei_PM1
Aspergillus_luchuensis_CBS_106.47	Aspergillus_awamori,Aspergillus_phoenicis_ATCC_13157
Blumeria_graminis_f._sp._tritici	Blumeria_graminis_f._sp._tritici_96224
Neurospora_tetrasperma_FGSC_2508	Neurospora_crassa,Neurospora_crassa_OR74A
Pseudogymnoascus_destructans_20631-21	Pseudogymnoascus_destructans
Aspergillus_pseudocaelatus	Aspergillus_tamarii
Aspergillus_flavus_AF70	Aspergillus_parasiticus_SU-1,Aspergillus_parasiticus
Saccharomyces_cerevisiae_YJM555	Saccharomyces_cerevisiae_AWRI1631,Saccharomyces_cerevisiae_S288C
Aspergillus_fumigatus_Z5	Aspergillus_fumigatiaffinis,Aspergillus_fischeri_NRRL_181
Metarhizium_rileyi_RCEF_4871	Metarhizium_rileyi
Aspergillus_sp._CLMG-2019a	Aspergillus_alliaceus
Pseudogymnoascus_sp._VKM_F-4516_(FW-969)	Pseudogymnoascus_sp._VKM_F-3808
Coccidioides_posadasii_C735_delta_SOWgp	Coccidioides_immitis_RMSCC_2394,Coccidioides_immitis
Trichophyton_mentagrophytes	Trichophyton_equinum_CBS_127.97,Trichophyton_tonsurans_CBS_112818
Rhynchosporium_commune	Rhynchosporium_agropyri
Alternaria_gaisen	Alternaria_sp._MG1
Bipolaris_sorokiniana_ND90Pr	Bipolaris_oryzae_ATCC_44560,Bipolaris_zeicola_26-R-13
Penicillium_solitum	Penicillium_nordicum
Pyrenophora_teres_f._maculata	Pyrenophora_teres_f._teres,Pyrenophora_teres_f._teres_0-1
Aspergillus_ochraceoroseus_IBT_24754	Aspergillus_ochraceoroseus
Pyrenophora_tritici-repentis_Pt-1C-BFP	Pyrenophora_tritici-repentis
Blumeria_graminis_f._sp._hordei_DH14	Blumeria_graminis_f._sp._hordei
Cordyceps_militaris_CM01	Cordyceps_militaris
Trichophyton_violaceum	Trichophyton_rubrum_CBS_735.88,Trichophyton_rubrum_CBS_289.86
Fonsecaea_multimorphosa_CBS_102226	Fonsecaea_multimorphosa
Fusarium_albosuccineum	Fusarium_decemcellulare
Sporothrix_schenckii_ATCC_58251	Sporothrix_brasiliensis_5110
Pseudogymnoascus_sp._VKM_F-4515_(FW-2607)	Pseudogymnoascus_sp._VKM_F-4517_(FW-2822)
Colletotrichum_asianum	Colletotrichum_gloeosporioides_Cg-14
Cryptococcus_gattii_WM276	Cryptococcus_gattii_E566,Cryptococcus_gattii_Ru294
Paracoccidioides_brasiliensis_Pb18	Paracoccidioides_brasiliensis,Paracoccidioides_brasiliensis_Pb03
Acidomyces_sp._'richmondensis'	Acidomyces_richmondensis_BFW
Sordaria_macrospora	Sordaria_macrospora_k-hell
Pochonia_chlamydosporia_170	Pochonia_chlamydosporia_123
Talaromyces_cellulolyticus	Talaromyces_verruculosus
Thermothielavioides_terrestris	Thermothielavioides_terrestris_NRRL_8126
Pseudogymnoascus_sp._VKM_F-103	Pseudogymnoascus_sp._VKM_F-4519_(FW-2642)
Botrytis_elliptica	Botryotinia_calthae,Botrytis_cinerea_BcDW1
Colletotrichum_orbiculare_MAFF_240422	Colletotrichum_sidae,Colletotrichum_trifolii
Tuber_melanosporum	Tuber_melanosporum_Mel28
Pseudogymnoascus_sp._05NY08	Pseudogymnoascus_sp._WSF_3629
Cryptococcus_neoformans_var._grubii_Gb118	Cryptococcus_neoformans_var._grubii_Br795,Cryptococcus_neoformans_var._grubii_C23
Beauveria_bassiana_ARSEF_2860	Beauveria_brongniartii_RCEF_3172
Hypoxylon_sp._CO27-5	Hypoxylon_sp._EC38
Tilletia_laevis	Tilletia_controversa
Trichoderma_lentiforme	Trichoderma_guizhouense,Trichoderma_harzianum_CBS_226.95
Pseudogymnoascus_sp._VKM_F-4518_(FW-2643)	Pseudogymnoascus_sp._VKM_F-4520_(FW-2644)
Fusarium_flagelliforme	Fusarium_coffeatum
Aspergillus_nomiae_NRRL_13137	Aspergillus_pseudonomius
Trichoderma_asperellum_CBS_433.97	Trichoderma_asperellum
Colletotrichum_fioriniae_PJ7	Colletotrichum_salicis
Letharia_lupina	Letharia_columbiana
Podospora_comata	Podospora_anserina_S_mat+
Fonsecaea_pedrosoi_CBS_271.37	Fonsecaea_nubica
Emmonsia_crescens	Emmonsia_crescens_UAMH_3008
Coleophoma_crateriformis	Coleophoma_cylindrospora
Pseudogymnoascus_sp._VKM_F-4513_(FW-928)	Pseudogymnoascus_sp._VKM_F-4246
Verticillium_dahliae_VDG2	Verticillium_nonalfalfae
Candida_albicans_P37039	Candida_albicans_P37005,Candida_albicans_P37037
Cryptococcus_gattii_VGII_CBS_10090	Cryptococcus_gattii_VGII_2001/935-1
Colletotrichum_tofieldiae	Colletotrichum_incanum
Colletotrichum_shisoi	Colletotrichum_tanaceti
Trichophyton_verrucosum_HKI_0517	Trichophyton_benhamiae_CBS_112371
Candida_albicans_P75063	Candida_albicans_GC75,Candida_albicans_P75016
Candida_albicans_P78042	Candida_albicans_P34048
Aspergillus_campestris_IBT_28561	Aspergillus_candidus
fungal_sp._No.14919	Xylaria_grammica
Aspergillus_uvarum_CBS_121591	Aspergillus_aculeatus_ATCC_16872,Aspergillus_aculeatinus_CBS_121060
Lachnellula_arida	Lachnellula_cervina
Rutstroemia_sp._NJR-2017a_WRK4	Rutstroemia_sp._NJR-2017a_BVV2
Cryptococcus_neoformans_var._grubii_Bt85	Cryptococcus_neoformans_var._grubii_Ze90-1
Histoplasma_capsulatum_H88	Histoplasma_capsulatum_H143
Ophiocordyceps_sinensis	Ophiocordyceps_sinensis_CO18
Aspergillus_terreus	Aspergillus_terreus_NIH2624
Saccharomyces_cerevisiae_VL3	Saccharomyces_cerevisiae_FostersB
Candida_albicans_P57072	Candida_albicans_P75010
Cladophialophora_carrionii_CBS_160.54	Cladophialophora_carrionii
Valsa_mali	Valsa_mali_var._pyri
Candida_albicans_12C	Candida_albicans_Ca529L,Candida_albicans_Ca6
Trichoderma_atroviride_IMI_206040	Trichoderma_gamsii
Candida_albicans_P94015	Candida_albicans_P60002
Cryptococcus_neoformans_var._grubii_125.91	Cryptococcus_neoformans_var._grubii_c45,Cryptococcus_neoformans_var._grubii_Bt63
Fusarium_vanettenii_77-13-4	Fusarium_sp._AF-4,Fusarium_euwallaceae,Fusarium_sp._AF-8
Yarrowia_lipolytica_CLIB122	Yarrowia_lipolytica
Ceratocystis_fimbriata_CBS_114723	Ceratocystis_platani
Aspergillus_glaucus_CBS_516.65	Aspergillus_ruber_CBS_135680
Penicillium_italicum	Penicillium_expansum
Zymoseptoria_brevis	Zymoseptoria_tritici_IPO323,Zymoseptoria_tritici_ST99CH_1A5
Aspergillus_sclerotioniger_CBS_115572	Aspergillus_carbonarius_ITEM_5010
Lachnellula_hyalina	Lachnellula_subtilissima
Kwoniella_mangroviensis_CBS_10435	Kwoniella_mangroviensis_CBS_8507

[1] Species less than 3% divergent at the protein sequence level were sampled to keep the longest representative (first column, filtered out species are listed in the second column).
