# Supplementary material for: Plant pathogens provide clues to the potential origin of bat white-nose syndrome Pseudogymnoascus destructans
Source: Virulence. 2022 Jun 6;13(1):1020–31. doi: 10.1080/21505594.2022.2082139 (PMC9176227; doi:10.1080/21505594.2022.2082139)
Supplement: Supplemental Material [file KVIR_A_2082139_SM3848.zip › supplementary/SupplementaryS1_8.25.pdf]

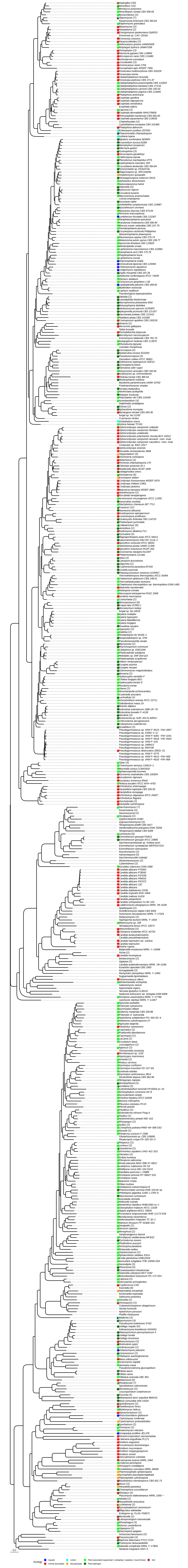

Conidiobolus coronatus NRRL 28638
Thamnocephalis sphaerospora
Syncephalis pseudoplumigaleata
Piptocephalis cylindrospora
Basidiobolus meristosporus CBS 931.73
Mucor (4)
Parasitella parasitica
Choanephora cucurbitarum
Rhizopus (4)
Phycomyces blakesleeanus NRRL 1555−−
Absidia (2)
Hesseltinella vesiculosa
Lichtheimia (2)
Syncephalastrum racemosum
Bifiguratus adelaidae
Endogone sp. FLAS−F59071
Mortierella (2)
Lobosporangium transversale
Rhizophagus (3)
Glomus cerebriforme
Gigaspora (2)
Diversispora epigaea
Schizosaccharomyces (5)
Pneumocystis (4)
Taphrina deformans PYCC 5710
Protomyces lactucaedebilis
Saitoella complicata NRRL Y−17804
Neolecta irregularis DAH−3
0.2
Ecology
Aquatic
Animal associated
Lichen
Mycoparasite
Plant associated (saprotroph / endophyte / epiphyte / mycorrhizae)
Plant pathogen
Soil
