# Supplementary material for: Plant pathogens provide clues to the potential origin of bat white-nose syndrome Pseudogymnoascus destructans
Source: Virulence. 2022 Jun 6;13(1):1020–31. doi: 10.1080/21505594.2022.2082139 (PMC9176227; doi:10.1080/21505594.2022.2082139)
Supplement: Supplemental Material [file KVIR_A_2082139_SM3848.zip › supplementary/_Supplemental TableS2.pdf]

Table S-2

Ancestral trait reconstruction under distinct models of character evolution (see Methods). Each entry is a probability computed from 1,000 stochastic mappings. ER: equal rate. SYM: symmetric model. ARD: all rates different. AVG: model average estimates, obtained by weighting each model by its AIC value. The reduced model contains only the four most frequently observed character states and was fitted to the corresponding data subset.

	Complete Model				Reduced Model			
Trait	ER	SYM	ARD	AVG	ER	SYM	ARD	AVG
Animal associated	2%	4%	7%	**4%**	3%	3%	8%	**8%**
Aquatic	1%	0%	1%	**0%**				
Lichen	0%	0%	1%	**0%**				
Mycoparasite	0%	0%	1%	**0%**				
Plant associated[1]	47%	40%	66%	**41%**	50%	51%	77%	**77%**
Plant pathogen	51%	56%	20%	**55%**	46%	45%	11%	**11%**
Soil	0%	0%	4%	**0%**	1%	0%	4%	**4%**
Plant associated + plant pathogen	98%	96%	86%	**96%**	96%	96%	88%	**88%**
AIC	1215.6	1015.7	1025.8		893.68	853.3	826.6	
AIC weights[2]	0.0000	0.9936	0.0064		0.0000	0.0000	0.9999	

Footnotes:

[1] Defined as a saprotroph, endophyte, epiphyte, or mycorrhizae

[2] AIC weights are computed as $w[i] = \exp(-1/2\ \Delta AIC[i]) / \mathrm{sum}(\exp(-1/2\ \Delta AIC[i]))$, where $\Delta AIC[i] = AIC[i] - \min(AIC)$
